# Supplementation of krill oil with high phospholipid content increases sum of EPA and DHA in erythrocytes compared with low phospholipid krill oil

**DOI:** 10.1186/s12944-015-0142-y

**Published:** 2015-11-04

**Authors:** V. R. Ramprasath, I. Eyal, S. Zchut, I. Shafat, P. J. H. Jones

**Affiliations:** Richardson Centre for Functional Foods and Nutraceuticals, University of Manitoba, 196 Innovation Drive, Winnipeg, MB R3T 2N2 Canada; Department of Human Nutritional Sciences, University of Manitoba, Winnipeg, Canada; Enzymotec Ltd. P.O.B 6, Migdal HaEmeq, Israel

**Keywords:** Krill oil, Phospholipids, EPA, DHA, Omega-3 index

## Abstract

**Background:**

Bioavailability of krill oil has been suggested to be higher than fish oil as much of the EPA and DHA in krill oil are bound to phospholipids (PL). Hence, PL content in krill oil might play an important role in incorporation of n-3 PUFA into the RBC, conferring properties that render it effective in reducing cardiovascular disease (CVD) risk. The objective of the present trial was to test the effect of different amounts of PL in krill oil on the bioavailability of EPA and DHA, assessed as the rate of increase of n-3 PUFA in plasma and RBC, in healthy volunteers.

**Methods and design:**

In a semi randomized crossover single blind design study, 20 healthy participants consumed various oils consisting of 1.5 g/day of low PL krill oil (LPL), 3 g/day of high PL krill oil (HPL) or 3 g/day of a placebo, corn oil, for 4 weeks each separated by 8 week washout periods. Both LPL and HPL delivered 600 mg of total n-3 PUFA/day along with 600 and 1200 mg/day of PL, respectively.

**Results:**

Changes in plasma EPA, DPA, DHA, total n-3 PUFA, n-6:n-3 ratio and EPA + DHA concentrations between LPL and HPL krill oil supplementations were observed to be similar. Intake of both forms of krill oils increased the RBC level of EPA (*p* < 0.001) along with reduced n-6 PUFA (LPL: *p* < 0.001: HPL: *p* = 0.007) compared to control. HPL consumption increased (*p* < 0.001) RBC concentrations of EPA, DPA, total and n-3 PUFA compared with LPL. Furthermore, although LPL did not alter RBC n-6:n-3 ratio or the sum of EPA and DHA compared to control, HPL intake decreased (*p* < 0.001) n-6:n-3 ratio relative to control with elevated (*p* < 0.001) sum of EPA and DHA compared to control as well as to LPL krill oil consumption. HPL krill oil intake elevated (*p* < 0.005) plasma total and LDL cholesterol concentrations compared to control, while LPL krill oil did not alter total and LDL cholesterol, relative to control.

**Conclusions:**

The results indicate that krill oil with higher PL levels could lead to enhanced bioavailability of n-3 PUFA compared to krill oil with lower PL levels.

**Trial registration:**

Clinicaltrials.gov# NCT01323036.

## Introduction

Marine n-3 polyunsaturated fatty acids (PUFA), including EPA and DHA, have been demonstrated to play beneficial roles in prevention of cardiovascular disease (CVD) [1, 2]. In addition to decreasing circulating lipid concentrations [3], n-3 PUFA have been found to reduce systolic blood pressure, inflammatory process and platelet aggregation as well as prevent arrhythmias [1, 4]. Consumption of n-3 PUFA including EPA and DHA occurs mainly through intake of oily fish or through supplements where the n-3 PUFA are usually esterified to glycerol of triglycerides (TG). However, other marine sources, such as krill oil, in which the n-3 PUFA are esterified to glycerol of phospholipids (PL), could also be used as source for n-3 PUFA. Consumption of n-3 PUFA esterified to PLs has been shown to exert a wide range of health benefits such as plasma lipid reduction [5], enhanced bioavailability of EPA and DHA [6–8] as well as anti-inflammatory [9] and antioxidant activity [10].

Krill oil has drawn a great interest as the PL-bound fatty acids are more efficiently absorbed and incorporated into cell membranes. Several animal and human studies have shown krill oil to be more efficient than fish oil in enhancing plasma and RBC n-3 PUFA and improving risk markers for CVD [5–8, 11, 12]. Studies have shown enhanced absorption of n-3 fatty acids into target organs such as heart, brain and liver when attached to PL compared to TG [9, 13, 14]. Benefits of krill over fish oil might be due to the structural differences between the two oils where the n-3 PUFA in krill and fish oil are esterified with PL and TG, respectively. The primary PL in krill oil is phosphatidylcholine (PC), and approximately 60–70 % of the omega-3 fatty acids in krill oil are bound to PLs.

Association between PL and long-chain n-3 PUFA facilitates the transfer of fatty acid molecules through the intestinal wall, increasing bioavailability and thereby improving the n-3:n-6 PUFA ratio [15]. As PL esterified n-3 PUFA are absorbed efficiently, amounts of PL present in the krill oil could play an important role in absorption, bioavailability and incorporation into different cellular compartments.

The objective of the present trial was to assess the bioavailability of krill oil consisting low PL content (LPL) compared to krill oil with high PL content (HPL), with similar n-3 PUFA content, and a placebo. The specific objective included assessment and comparison of fatty acid composition in plasma as well as incorporation into red blood cells of volunteers at baseline and following 4 weeks of intervention with each of the oils, each separated by an 8 week washout period. Another objective was to measure lipid profiles including total cholesterol, HDL, LDL, and TG levels following 4 weeks consumption of LPL or HPL krill oil.

## Results

Twenty out of 24 participants who completed the first three phases continued and completed the study for the 4^th^ phase with LPL treatment. Vital signs including body weight, systolic and diastolic blood pressure and heart rate were within normal ranges (Table 1). Physical examination by the physician revealed that none of the participants had any health problems or concerns known to affect study outcomes. The study physician recorded and compared the health status of participants at each visit. None of the participants during the study showed major changes in their health status related to the trial.

**Table 1:** Anthropometric measures of participants during HPL and LPL krill and control oil interventions

Parameter	Treatment	Baseline	Endpoint	Change (Difference)
Body Weight (Kg)	HPL Krill oil	67.53 ± 2.75	67.96 ± 2.63	0.43 ± 0.37
	LPL krill oil	67.99 ± 2.77	67.74 ± 2.73	−0.25 ± 0.22^a^
	Control	67.56 ± 2.46	67.73 ± 2.44	0.18 ± 0.22
BMI (kg/m^2^)	HPL Krill oil	23.62 ± 0.68	23.75 ± 0.63	0.13 ± 0.15
	LPL krill oil	23.79 ± 0.71	23.69 ± 0.71	−0.10 ± 0.08
	Control	23.61 ± 0.63	23.67 ± 0.62	0.06 ± 0.09
Waist (cm)	HPL Krill oil	77.95 ± 1.98	78.20 ± 1.93	0.25 ± 0.27
	LPL krill oil	78.25 ± 2.03	77.81 ± 2.05	−0.43 ± 0.37
	Control	78.05 ± 1.80	78.14 ± 1.78	0.09 ± 0.15
Hip (cm)	HPL Krill oil	92.04 ± 1.19	92.45 ± 1.14	0.41 ± 0.34
	LPL krill oil	92.69 ± 1.22	92.92 ± 1.20	0.23 ± 0.28
	Control	92.70 ± 1.10	92.71 ± 1.07	0.01 ± 0.12
Waist/Hip ratio	HPL Krill oil	0.85 ± 0.02	0.84 ± 0.02	0.00 ± 0.00
	LPL krill oil	0.84 ± 0.02	0.84 ± 0.02	−0.01 ± 0.00
	Control	0.84 ± 0.02	0.84 ± 0.01	0.00 ± 0.00
Systolic BP (mmHg)	HPL Krill oil	106.80 ± 2.63	108.14 ± 2.00^a^	1.35 ± 1.39
	LPL krill oil	106.35 ± 2.45	103.97 ± 2.73	−2.38 ± 1.56
	Control	107.99 ± 2.13	105.89 ± 2.36	−2.11 ± 1.15
Diastolic BP (mmHg)	HPL Krill oil	71.45 ± 1.69	72.53 ± 1.32	1.09 ± 0.88
	LPL krill oil	71.17 ± 1.96	70.5 ± 1.86	−0.67 ± 1.34
	Control	72.26 ± 1.68	71.05 ± 1.84	−1.21 ± 1.05
Heart rate (Beats/min)	HPL Krill oil	71.35 ± 2.43	70.25 ± 1.72	−1.10 ± 2.07
	LPL krill oil	73.25 ± 2.23	69.1 ± 1.74	−4.15 ± 1.70
	Control	71.55 ± 1.66	71.80 ± 1.65	0.25 ± 1.09

Values are expressed as Mean ± SEM. *n* = 20. ^a^denotes significance compared with LPL krill oil intervention

A weekly telephone questionnaire was completed by the study coordinator to monitor compliance. Questions included capsule intake, fish or sea food intake, adverse events and concomitant medication use. No adverse or serious adverse events related to the treatments were reported during the period of the study. During the study one participant experienced a minor stomach ulcer which, with treatment by a physician, was resolved in two weeks. The event occurred only during the washout period and was deemed not related to the study by the study physician. None of the participants consumed any concomitant medications or supplements that are known to affect study outcomes. All participants followed the guidelines not to eat more than one serving per month of fish or sea food. None of the participants exceeded the limit of fish or sea food consumption during intervention or washout periods. No differences with the gastrointestinal responses were observed between the LPL and HPL krill oil and placebo consumptions.

Participants consumed the study capsules regularly according to instructions provided. According to the self-reported capsule consumption checklist each participant completed, participants showed high compliance with capsule consumption. The compliance ratings for capsule consumption during LPL krill oil was 94.6 % compared to 95.8 % for HPL krill oil and 97.4 % for placebo interventions.

The safety with consumption of LPL krill was determined by measuring blood parameters including WBC, RBC, hematocrit, hemoglobin, and platelet count, neutrophils, lymphocytes, monocytes, eosinophils, basophils, MCV, MCH and MCHC values. No significant differences were found in the above parameters between consumption of placebo or LPL or HPL krill oil (Data not shown).

Anthropometric measures were performed at the beginning and end of each phase. At baseline, 16 participants were of BMI < 25 and 8 participants had a BMI between 25 and 28. No significant changes were observed in any of the anthropometric measures between different treatments phases except for a decrease (*p* = 0.033) in body weight following consumption of LPL, relative to HPL (Table 1).

Consumption of HPL krill oil elevated the plasma concentrations of EPA (*p* < 0.001), DPA (*p* = 0.015), DHA (*p* < 0.001), n-3 PUFA (*p* < 0.001) and EPA + DHA (*p* < 0.001) relative to control. No significant change in total PUFA was observed compared to control (Table 2). Similarly, LPL krill oil increased (*p* < 0.001) the plasma concentrations of EPA, DPA, DHA, n-3 PUFA and EPA + DHA. Both LPL and HPL krill oils reduced n-6:n-3 ratio (*p* < 0.001), while only LPL krill oil reduced n-6 PUFA (*p* = 0.031) compared to control.

**Table 2:** Plasma fatty acids concentrations during each of the experimental phases

Fatty acids (%)	Treatment	Baseline	Endpoint	Change (Difference)
C20:5n-3 Eicosapentaenoic acid (EPA)	HPL Krill oil	0.79 ± 0.07	2.05 ± 0.12^a^	1.26 ± 0.11^a^
	LPL krill oil	0.76 ± 0.06	1.98 ± 0.11^a^	1.23 ± 0.13^a^
	Control	0.88 ± 0.08	0.76 ± 0.10	−0.12 ± 0.07
C22:5n-3 Docosapentaenoic acid (DPA)	HPL Krill oil	0.63 ± 0.07	0.78 ± 0.06^ab^	0.15 ± 0.05^a^
	LPL krill oil	0.76 ± 0.04	0.98 ± 0.05^a^	0.22 ± 0.04^a^
	Control	0.65 ± 0.06	0.61 ± 0.04	−0.04 ± 0.05
C22:6n-3 Docosahexaenoic acid (DHA)	HPL Krill oil	2.97 ± 0.13	3.99 ± 0.19^ab^	1.01 ± 0.15^a^
	LPL krill oil	3.06 ± 0.12	4.35 ± 0.22^a^	1.29 ± 0.13^a^
	Control	3.15 ± 0.14	2.90 ± 0.13	−0.25 ± 0.16
Total SFA	HPL Krill oil	43.36 ± 0.44	42.80 ± 0.35^b^	−0.57 ± 0.45^b^
	LPL krill oil	44.99 ± 0.66	41.83 ± 0.34	−3.16 ± 0.72^a^
	Control	41.69 ± 1.11	42.49 ± 0.37	0.79 ± 1.12
Total MUFA	HPL Krill oil	17.58 ± 0.68	16.44 ± 0.57	−1.13 ± 0.68^b^
	LPL krill oil	12.73 ± 1.31	15.65 ± 0.47^a^	2.92 ± 1.30
	Control	17.63 ± 0.70	16.96 ± 0.51	−0.67 ± 0.68
Total PUFA	HPL Krill oil	39.06 ± 0.54	40.76 ± 0.44^b^	1.70 ± 0.55
	LPL krill oil	42.27 ± 0.77	42.52 ± 0.47^a^	0.25 ± 0.77
	Control	40.68 ± 0.94	40.56 ± 0.46	−0.12 ± 1.12
Total n-6 PUFA	HPL Krill oil	34.04 ± 0.54	33.36 ± 0.45^a^	−0.68 ± 0.43^b^
	LPL krill oil	37.09 ± 0.77	34.61 ± 0.59	−2.48 ± 0.67^a^
	Control	35.40 ± 0.86	35.70 ± 0.42	0.30 ± 0.96
Total n-3 PUFA	HPL Krill oil	5.02 ± 0.16	7.40 ± 0.28^a^	2.38 ± 0.24^a^
	LPL krill oil	5.19 ± 0.16	7.91 ± 0.32^a^	2.72 ± 0.27^a^
	Control	5.28 ± 0.21	4.86 ± 0.15	−0.42 ± 0.20
n-6:n-3	HPL Krill oil	6.93 ± 0.27	4.69 ± 0.26^a^	−2.25 ± 0.23^a^
	LPL krill oil	7.29 ± 0.29	4.58 ± 0.27^a^	−2.72 ± 0.19^a^
	Control	6.87 ± 0.28	7.46 ± 0.23	0.59 ± 0.17
EPA + DHA	HPL Krill oil	3.76 ± 0.15	6.04 ± 0.28^a^	2.27 ± 0.22^a^
	LPL krill oil	3.82 ± 0.14	6.33 ± 0.30^a^	2.51 ± 0.23^a^
	Control	4.03 ± 0.19	3.66 ± 0.13	−0.37 ± 0.18

Values are expressed as Mean ± SEM. *n* = 20. ^a^ and ^b^ denote significance compared with control or LPL krill oil interventions respectively

Comparing the change between HPL and LPL showed no differences in plasma EPA, DPA, DHA, total PUFA, total n-3, EPA + DHA and n-6:n-3 ratio following consumption of the two oils (Table 2). However, LPL krill oil intake reduced the SFA (*p* = 0.035) and n-6 PUFA (*p* = 0.040) and increased MUFA (*p* = 0.037) in plasma compared with HPL krill oil (Table 2).

Intake of HPL krill oil significantly increased RBC concentrations of EPA (*p* < 0.001), DHA (*p* = 0.017), EPA + DHA (the omega-3 index, *p* < 0.001) and n-3 PUFA (*p* < 0.001) compared to control. HPL krill oil consumption decreased n-6 PUFA (*p* = 0.007) and n-6:n-3 ratio (*p* = 0.021), with no significant difference in DPA, and total PUFA levels, compared to control (Table 3).

**Table 3:** RBC fatty acids concentrations during each of the experimental phases

Fatty acids (%)	Treatment	Baseline	Endpoint	Change (difference)
C20:5n-3 Eicosapentaenoic acid (EPA)	HPL Krill oil	0.82 ± 0.05	1.51 ± 0.09^ab^	0.69 ± 0.06^ab^
	LPL krill oil	0.66 ± 0.04	1.09 ± 0.06^a^	0.43 ± 0.06^a^
	Control	0.83 ± 0.05	0.73 ± 0.05	−0.09 ± 0.03
C22:5n-3 Docosapentaenoic acid (DPA)	HPL Krill oil	2.26 ± 0.06	2.42 ± 0.05^b^	0.16 ± 0.04^b^
	LPL krill oil	1.68 ± 0.12	1.65 ± 0.13^a^	−0.03 ± 0.14
	Control	2.46 ± 0.15	2.53 ± 0.20	0.07 ± 0.24
C22:6n-3 Docosahexaenoic acid (DHA)	HPL Krill oil	4.21 ± 0.18	4.55 ± 0.17^ab^	0.34 ± 0.09^a^
	LPL krill oil	3.26 ± 0.21	3.14 ± 0.21	−0.12 ± 0.27
	Control	3.70 ± 0.39	3.71 ± 0.31	0.02 ± 0.41
Total SFA	HPL Krill oil	43.71 ± 0.39	43.25 ± 0.41^b^	−0.45 ± 0.43^b^
	LPL krill oil	46.87 ± 0.81	48.98 ± 0.73^a^	2.11 ± 0.95^a^
	Control	44.03 ± 0.40	43.71 ± 0.36	−0.32 ± 0.43
Total MUFA	HPL Krill oil	18.11 ± 0.27	18.55 ± 0.52^b^	0.44 ± 0.49
	LPL krill oil	17.05 ± 0.26	17.25 ± 0.26^a^	0.20 ± 0.27
	Control	18.48 ± 0.33	18.30 ± 0.29	−0.18 ± 0.28
Total PUFA	HPL Krill oil	38.18 ± 0.54	38.20 ± 0.46^b^	0.01 ± 0.43^b^
	LPL krill oil	36.09 ± 0.81	33.77 ± 0.81^a^	−2.31 ± 0.87^a^
	Control	37.49 ± 0.53	37.99 ± 0.52	0.50 ± 0.61
Total n-6 PUFA	HPL Krill oil	30.55 ± 0.46	29.38 ± 0.44^ab^	−1.17 ± 0.36^a^
	LPL krill oil	30.20 ± 0.58	27.59 ± 0.54^a^	−2.61 ± 0.50^a^
	Control	30.18 ± 0.49	30.69 ± 0.43	0.51 ± 0.52
Total n-3 PUFA	HPL Krill oil	7.63 ± 0.22	8.81 ± 0.25^ab^	1.18 ± 0.15^ab^
	LPL krill oil	5.88 ± 0.31	6.18 ± 0.36^a^	0.30 ± 0.40
	Control	7.31 ± 0.38	7.30 ± 0.23	−0.01 ± 0.29
n-6:n-3	HPL Krill oil	4.08 ± 0.14	3.41 ± 0.14^ab^	−0.67 ± 0.07^a^
	LPL krill oil	5.36 ± 0.25	4.73 ± 0.27	−0.64 ± 0.24
	Control	4.51 ± 0.41	4.29 ± 0.15	−0.22 ± 0.32
EPA + DHA	HPL Krill oil	5.03 ± 0.21	6.06 ± 0.24^ab^	1.03 ± 0.11^ab^
	LPL krill oil	3.92 ± 0.23	4.23 ± 0.26	0.31 ± 0.28
	Control	4.53 ± 0.40	4.45 ± 0.32	−0.08 ± 0.42

Values are expressed as Mean ± SEM. *n* = 20. ^a^ and ^b^ denote significance compared with control or LPL krill oil interventions respectively

LPL krill oil supplementation increased EPA concentration in the RBC compared to control (*p* < 0.001). Interestingly, LPL krill oil consumption failed to change the RBC concentrations of DPA, DHA, n-6:n-3 ratio and EPA + DHA compared to control. LPL krill oil reduced total PUFA (*p* < 0.025) and total n-6 PUFA (*p* < 0.001) concentrations in RBC compared to control. Importantly, consumption of HPL krill oil led to elevations in RBC concentrations of EPA (*p* < 0.001), DPA (*p* < 0.040), total PUFA (*p* < 0.030), total n-3 PUFA (*p* < 0.040) and EPA + DHA (*p* < 0.020), compared to LPL (Table 3).

No significant changes were found in serum lipid concentrations and ratio between total and HDL cholesterol of participants during the ingestion of LPL krill compared to HPL krill oil (Table 4). However, HPL krill oil supplementation elevated serum total (*p* = 0.024) and LDL cholesterol concentrations (*p* < 0.001) compared to control.

**Table 4:** Serum lipid concentrations during the study

Parameters (mMol/L)	Treatment	Baseline	Endpoint	Change (Difference)
TG	HPL Krill oil	98.02 ± 11.76	94.90 ± 9.25^b^	−3.12 ± 5.50
	LPL krill oil	106.2 ± 9.62	101.21 ± 10.41	−4.99 ± 4.74
	Control	99.91 ± 10.02	101.51 ± 10.93	1.60 ± 4.78
HDL	HPL Krill oil	54.52 ± 3.14	59.36 ± 3.25	4.84 ± 1.75
	LPL krill oil	55.48 ± 3.33	57.81 ± 3.76	2.33 ± 1.91
	Control	54.71 ± 3.02	56.65 ± 2.90	1.94 ± 1.12
LDL	HPL Krill oil	94.28 ± 5.47	101.52 ± 7.00^a^	7.25 ± 3.67^a^
	LPL krill oil	96.93 ± 7.25	100.32 ± 8.27^a^	3.40 ± 3.35
	Control	93.89 ± 5.07	92.30 ± 5.70	−1.60 ± 2.79
TC	HPL Krill oil	168.02 ± 6.26	179.61 ± 7.65^a^	11.59 ± 4.19^a^
	LPL krill oil	173.62 ± 7.06	177.68 ± 9.18	4.06 ± 4.76
	Control	169.56 ± 5.53	169.56 ± 6.06	0.00 ± 3.62
TC/HDL	HPL Krill oil	3.28 ± 0.23	3.22 ± 0.26	1.50 ± 0.42
	LPL krill oil	3.41 ± 0.31	3.34 ± 0.30	1.93 ± 0.58
	Control	3.32 ± 0.22	3.21 ± 0.23	1.96 ± 0.74

Values are expressed as Mean ± SEM. *n* = 20. ^a^ and ^b^ denote significance compared with control and LPL respectively. TG- triglycerides, *HDL* high density lipoprotein, *LDL* low density lipoprotein, *TC* total cholesterol

## Discussion

In the present study, consumption of HPL and LPL krill oil exhibited similar effects on plasma fatty acids profile but different actions on RBC fatty acid concentrations. Results suggest that the absorption of omega-3 fatty acids in plasma is less affected by the amount of PLs, as the level of EPA, DHA, DPA and total n-3 PUFA, while increasing compared to control, did not consistently change according to PL levels (Table 2). Nonetheless, incorporation of absorbed n-3 PUFAs into the membranes of RBC appears to depend on the amount of PL in the test product used. Results show that levels of EPA, DPA, total n-3 PUFA and EPA + DHA in RBC were increased following HPL krill oil consumption compared to LPL krill oil (Table 3). The difference between absorption of omega-3 fatty acids into plasma and into RBC may be significant relating to the fate of these fatty acids. While plasma levels of EPA and DHA are considered a marker of short term absorption, easily affected by levels of omega-3 in the supplement, their levels in RBC are markers of long term absorption as well as of tissue absorption of these fatty acids [16, 17]. The exact mechanism by which PL influence the incorporation of n-3 PUFA into RBC is not completely understood and needs to be further investigated.

PLs are well recognised for their role in structure and function of cell membranes as well as formation of lipoprotein, and transportation and delivery of lipids to various tissues through the blood [18]. PLs are amphiphilic in nature and hence possess emulsifying properties. PLs are capable of modifying the surface structure of ingested fats which help pancreatic lipases to cleave long chain fatty acids [19]. In addition, PLs help in mixed micelle formation and enhance lipid absorption. In humans, dietary PLs are hydrolysed to lysoPC and free fatty acid (FFA) which are then absorbed through the enterocytes or after binding to albumin, a process which increases the delivery of PUFAs to tissues [20, 21]. Studies in animals suggest that up to 20 % of the may also be absorbed intact, without being first cleaved by PLA2 [22–25]. Studies have also shown that lysoPC could preferentially be a vehicle to deliver DHA to the brain [26, 27]. DHA is transported in plasma as either non esterified or lysoPC forms. Non esterified form of DHA gets delivered to platelets, whereas lysoPC form is delivered to RBC [28]. In both humans and mice, there was a proportional association between plasma and dietary EPA, whereas saturable incorporation of DHA into plasma lipids was found [29, 30]. Mechanisms by which the fatty acids are taken by the cells involves carrier mediated as well as passive transmembrane translocation [31, 32]. Hence, concentration of PL might have influenced the incorporation of n-3 PUFA into the RBC in the current study.

Intake of <300 mg/d of DHA by humans have been previously shown not to change the RBC concentrations of DHA [33, 34]. Dosage of DHA with high or low PL in the current study is around 200 mg/d which is comparable to dosage of these previous reports. In addition, Ramprasath et al. have previously shown that krill oil is more efficient than fish oil in increasing the EPA and DHA levels in plasma as well as RBC [8]. Similarly, others [6, 7, 11] have also reported similar findings of higher efficiency with krill oil compared to fish oil in raising circulating n-3 PUFA levels. Hence, PL-bound EPA and DHA from krill oil might be absorbed more efficiently than TG- or EE-bound EPA and DHA from fish oil. Based on the findings of the current study, along with previous studies, it appears that PL may play a vital role in incorporation of EPA and DHA into the RBC. However, understanding better the exact mechanism is of importance.

Consumption of EPA and DHA is well established to decrease levels of plasma TG, however, it is also known to elevate levels of LDL-C and TC [35]. Both LPL and HPL krill oil intakes elevated total and LDL cholesterol concentrations. An important parameter used as a risk factor for CVD is the ratio of TC/HDL-C. This ratio did not change in the current study, suggesting that TC and LDL-C increase might not be detrimental. These results support findings from our previous study which demonstrated an incremental increase [8], as well as other studies showing no change [6, 7, 36], in plasma concentrations of total and LDL cholesterol in normolipidemic participants after krill oil supplementation. Both LPL and HPL krill oils were well tolerated by the participants and caused no adverse effects which shows the safety nature of krill oil.

The current study involved a healthy population which allowed studying the bioavailability of krill oil with different amounts of PL on the levels of omega-3 PUFA in the plasma as well as RBC; thus we could expect more significant health benefits when consumed by individuals with metabolic syndrome or increased risk for CVD. High compliance by the participants to the study protocol including the intake of supplements as well as restrictions with fish, sea foods and supplements further added more robustness to the results. A crossover design helps to eliminate any variances due to genetic background of participants.

Although the study was a placebo controlled cross over study, the design was a semi randomised where the LPL phase was added at the end of the trial and not randomised. Same dose of n-3 PUFA/day was administered to the participants with two levels of doses of PL which is actually strength of the study. However, having only 3 capsules for the LPL phase, compared to 6 capsules for the HPL and control phases, affects the double blinding nature of the study.

## Experimental design

### Participant selection

Healthy males and non-pregnant females (*n* = 24; 12 males and 12 females) ages 18–49 years were recruited by advertisement at the Richardson Centre for Functional Foods and Nutraceuticals on the University of Manitoba campus. Participants were excluded if they have any chronic disease or admitted an allergy to fish or sea foods, or reported consuming supplements including n-3 PUFA in the past 6 months or consuming more than one fish serving per month during the month prior to the start of the study. Participants were included if they were nonsmokers; serum TG levels <200 mg/dL; total cholesterol <240 mg/dL; LDL-cholesterol <160 mg/ dL; BMI < 28, not consuming more than one alcoholic drink/day and not taking any medications that would interfere with lipid metabolism or to control blood lipids or treat hypertension.

### Ethics, consent and permissions

The protocol of the study was reviewed and approved by the Human Ethical Review Committee of the University of Manitoba (reference #B2011:014). All participants were explained the study protocol and written consent was obtained to participate in the study along with consent for publication of the data.

### Study design and intervention

A double blinded, randomized, placebo-controlled, crossover trial with 24 healthy participants was completed [8]. The completed study was comprised of treatments including identical looking capsules consisting of krill oil or an oil with high fish oil content each providing 600 mg of n-3 PUFA or a placebo, corn oil. Duration of each treatment phase was 4 weeks separated by washout phases of 8 weeks.

At the end of the study, participants were invited to continue the study for another single blind non-randomized phase for 12 weeks (8 washout weeks followed by 4 treatment weeks). Participants, whom continued to the fourth phase (*n* = 20; 11 males and 9 females), (Baseline BMI for 14 participants was <25 and BMI for 6 participants were between 25 and 28) received three capsules per day consisting of LPL krill oil providing 600 mg of n-3 PUFA (for comparison of the oils intake and composition see Tables 5 and 6, respectively). LPL was HP K•REAL™ and HPL was K•REAL™ pure krill oil, both by Enzymotec Ltd. Data obtained with LPL krill phase were compared with those from control and HPL krill oil phases. As the fish oil phase was not needed for the current objective, data from the fish oil phase were not used in the current investigation. All the rest of the human and laboratory experimental methods used were same as performed during the first three phases [8], as follows.

**Table 5:** Daily amounts of EPA, DHA, n-3 and n-6 PUFA in the supplements of the various study groups

Fatty acid (mg/day)	Corn oil	HPL Krill oil	LPL Krill oil
EPA	0	337	320
DHA	0	188	207
total n-3 PUFA	30	603	608
total n-6 PUFA	1437	60	34

**Table 6:** Fatty acid composition of HPL krill oil, LPL krill oil and corn oil used in the study

Fatty acid (% of total fatty acids)	HPL Krill oil	LPL Krill oil	Corn oil
C12 (Lauric)	0.27	0	0.13
C14 (Myristic)	11.82	2.33	0.00
C14:1 (Myristoleic)	0.16	0	0.00
C15 (Pentadecanoic)	0.47	0.18	0.00
C16 (Palmitic)	22.11	12.56	15.01
C16:1 (Palmitoleic)	6.09	2.19	0.00
C17 (Margaric)	2.02	0.17	0.00
C18 (Stearic)	1.39	2.39	3.31
C18:1n9 (Oleic)	13.28	7.89	1.08
C18:1n7 (Vaccenic)	7.56	4.30	0.00
C18:2n6 (Linoleic)	2.06	1.57	77.57
C18:3n6 (gamma-Linolenic)	0.00	0.25	0.00
C18:3n3 (alpha-Linolenic)	0.93	0.96	1.61
C18:4n3 (Moroctic)	2.70	1.78	0.00
C20 (Arachidic)	0.00	0.25	0.57
C20:1n9 (Eicosenoic)	0.80	1.01	0.44
C20:2n6 (Eicosadienoic)	0.41	0.20	0.00
C20:4n6 (Arachidonic)	0.40	1.40	0.00
C20:4n3 (Eicosatetraenoic)	0.26	1.1	0.00
C20:5n3 (Eicosapentaenoic)	16.44	31.92	0.00
C21:5n3 (Docosapentaenoic)	0	1.14	0
C22 (Behenic)	0.00	0	0.27
C22:1n11 (Cetoleic)	0	0.82	0
C22:1n9 (Euricic)	0.56	0.72	0.00
C22:2n9 (Docosadienoic)	0.43	0	0.00
C22:5n6 (Docosapentaenoic)	0	0.42	0
C22:5n3 (Docosapentaenoic)	0.38	3.07	0.00
C22:6n3 (Docosahexaenoic)	9.48	20.53	0.00
C24:1n9 (Nervonic)	0.00	0.86	0.00
Total n-3 PUFA	30.18	60.49	1.61
Total n-6 PUFA	2.86	3.84	77.57
Total n-9 PUFA	15.07	10.48	1.52
Total PUFA	33.47	64.33	79.18
Total MUFA	28.46	17.80	1.52
Total SFA	38.07	17.88	19.30

### Anthropometric measurements

Anthropometric and blood pressure measurements as well as physical examinations were performed at baseline and endpoint of each treatment following standard procedures [37].

### Plasma and RBC fatty acid analyses

Fasting blood samples were collected at baseline and endpoint of each phase. Samples were centrifuged at 3000 rpm for 20 min followed by separation of plasma and RBC and aliquots were stored in −80 ° C until analysis.

Plasma and RBC total lipids were extracted using the Folch method [38] which involved chloroform-methanol (2:1, v/v) containing 0 · 01 % BHT (Sigma-Aldrich, Oakville, ON, Canada) and heptadecanoic acid as an internal standard (Sigma-Aldrich, Oakville, ON, Canada). Extracted fatty acids were methylated with methanolic HCl. Fatty acid methyl esters were separated on a Supelcowax 10 column (30 m X 0 · 25 mm with 0 · 25 mm film thickness; Supelco, Bellefonte, PA, USA) using a gas chromatograph equipped with a flame ionisation detector (Bruker 430). Individual fatty acids were identified by comparison with known standards (NuChek Prep, Inc., Elysian, MN, USA). Individual fatty acids were calculated according to the peak area relative to the total area and expressed as the percentage of total fatty acids [8].

Serum lipid profiles including total and HDL-cholesterol and triglyceride levels were measured using a Vitros 350 Autoanalyser (Orthoclinical diagnostics). LDL-cholesterol levels were calculated using Friedewald equation [39]. Complete blood counts were also determined using a Beckmann coulter LH780 at baseline and at endpoint of each treatment phase.

### Statistical analysis

Control, HPL and LPL krill oil treatment phases were compared to each other. Differences between LPL and HPL krill oil and placebo treatments were analyzed for each dependent measure. Normal distribution of the variables was tested by Shapiro Wilkes test. Variables were analyzed by two-tailed paired student’s *t*-test for normally distributed variables or by Wilcoxon’s signed rank test for non-parametric variables. Values were expressed as mean ± SEM and p values < 0.05 were considered significant. Safety data were examined descriptively for each arm and compared across treatments.

## Abbreviations

CVD: Cardiovascular disease
DHA: Docosahexaenoic acid
EPA: Eicosapentaenoic acid
HDL: High-Density Lipoproteins
HPL: High phospholipid
LDL: Low-Density Lipoproteins
LPL: Low phospholipid
PUFA: Poly unsaturated fatty acids
